# The RASopathies: from pathogenetics to therapeutics

**DOI:** 10.1242/dmm.049107

**Published:** 2022-02-18

**Authors:** Katie E. Hebron, Edjay Ralph Hernandez, Marielle E. Yohe

**Affiliations:** Pediatric Oncology Branch, Center for Cancer Research, National Cancer Institute, Bethesda, MD 20892, USA

**Keywords:** Cardiofaciocutaneous syndrome, Costello syndrome, Legius syndrome, Noonan syndrome, RAS, RASopathies

## Abstract

The RASopathies are a group of disorders caused by a germline mutation in one of the genes encoding a component of the RAS/MAPK pathway. These disorders, including neurofibromatosis type 1, Noonan syndrome, cardiofaciocutaneous syndrome, Costello syndrome and Legius syndrome, among others, have overlapping clinical features due to RAS/MAPK dysfunction. Although several of the RASopathies are very rare, collectively, these disorders are relatively common. In this Review, we discuss the pathogenesis of the RASopathy-associated genetic variants and the knowledge gained about RAS/MAPK signaling that resulted from studying RASopathies. We also describe the cell and animal models of the RASopathies and explore emerging RASopathy genes. Preclinical and clinical experiences with targeted agents as therapeutics for RASopathies are also discussed. Finally, we review how the recently developed drugs targeting RAS/MAPK-driven malignancies, such as inhibitors of RAS activation, direct RAS inhibitors and RAS/MAPK pathway inhibitors, might be leveraged for patients with RASopathies.

## Abstract (precis)

**Summary:** RASopathies are developmental disorders caused by germline pathogenic variants in RAS/MAPK pathway genes. Here, we review the preclinical studies that provide the basis for future interventional clinical trials for RASopathy patients.

## Introduction

The RASopathies are a clinically defined group of disorders caused by a germline mutation in one of the genes encoding components of the RAS/MAPK pathway (Rauen, 2013; Tidyman and Rauen, 2009), typically resulting in increased signaling through this pathway. The RAS/MAPK pathway is a signal transduction cascade that is central to such normal cellular processes as proliferation, survival, differentiation and metabolism. This pathway is essential for normal development, with knockout mouse models of several components resulting in embryonic lethality due to, in many cases, abnormal vascularization of the embryo and/or placenta (Table S1). The pathway is composed of the RAS proteins, RAS guanine nucleotide exchange factors (GEFs), RAS GTPase-activating proteins (GAPs), RAS effector proteins and their targets, and other pathway modulators.

The specific classification of RASopathies is a matter of some debate among experts (Gross et al., 2020a). However, the RASopathies are generally accepted to include neurofibromatosis type 1 (NF1), Noonan syndrome (NS), Noonan syndrome with multiple lentigines (NS-ML), cardiofaciocutaneous syndrome (CFC), Costello syndrome (CS), Legius syndrome (LS), central conducting lymphatic anomalies syndrome (CCLA), SYNGAP1 syndrome, and capillary malformation arteriovenous malformation syndrome (CM-AVM). Many of the RASopathies have overlapping clinical features; however, each syndrome also has its unique characteristics. The overlapping features can include short stature, dysmorphic facial features, congenital heart disease, lymphatic dysfunction and intellectual disability. RASopathies are a common cause of non-immune hydrops fetalis (Sparks et al., 2020), as well as other features that can be observed on prenatal ultrasound such as increased nuchal translucency, cystic hygroma, congenital heart disease or pleural effusions (Scott et al., 2021). Each of the RASopathies are single-gene inheritance disorders. NS, CFC, CCLA and CM-AVM can be caused by alterations in one of several genes, while NF1, NS-ML, CS, LS and SYNGAP1 syndrome are each linked to alterations in one specific gene.

**Fig. 1.**
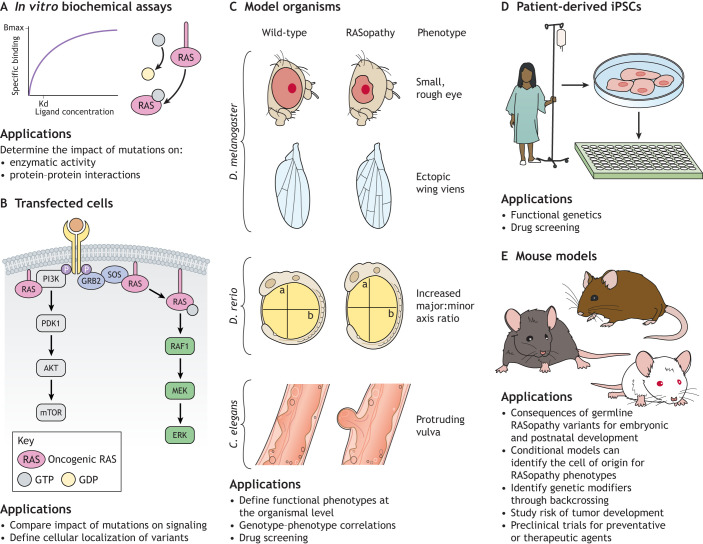
**Modeling the RASopathies.** (A) Biochemical assays with purified proteins. These assays can compare the impacts of RASopathy-associated mutations on the enzymatic activities of the chosen proteins. Some examples include kinase, phosphatase, GEF-stimulated exchange, GEF-independent exchange, GAP-stimulated hydrolysis and GAP-independent hydrolysis activities. In addition, purified protein can be used to determine the impact of RASopathy variants on protein–protein interactions. Bmax, maximum specific binding; Kd, dissociation constant. (B) Transfection of RASopathy variants into mammalian cells. These assays assess the ability of the variants to stimulate the RAS/MAPK pathway, as well as define the subcellular localization of the variants in comparison to the wild-type protein. (C) Expression of RASopathy variants in model organisms. These models facilitate the study of the impact of RAS/MAPK pathway activation at the organismal level. In *Drosophila melanogaster*, aberrant RAS/MAPK signaling in the eye leads to small, rough eyes, while aberrant signaling in the wing leads to ectopic wing development. Similarly, aberrant RAS/MAPK signaling in the zebrafish (*Danio rerio*) embryo leads to elongated embryos with an increased major:minor axis ratio, and aberrant RAS/MAPK signaling in developing *Caenorhabditis elegans* embryos leads to various vulvar phenotypes. These phenotypes can be quantified, elucidating the genotype/phenotype correlations when comparing RASopathy-associated variants in these systems. The *D. melanogaster*, *D. rerio*, *C. elegans* and *Mus musculus* orthologs of each of the human RASopathy-associated genes are listed in Table S2. (D) Patient-derived cell lines. Fibroblast lines, lymphoblastoid lines and induced pluripotent stem cells can be used in a variety of cell signaling and phenotypic assays, provided an appropriate control line is available for comparison. Model organisms and patient-derived cell lines can both be used for preclinical drug screening. (E) Knock-in/knockout mouse models. These can be used to determine the developmental stage at which a variant can trigger the RASopathy phenotype, as well as identify the cell of origin for the RASopathy phenotypes. Germline expression of RASopathy variants in mouse models can identify genetic modifiers of the phenotype through backcrossing onto different genetic backgrounds. Moreover, these mouse models can be used in preclinical studies of potential therapeutics. Knockout models of RASopathy genes are summarized in Table S1, while knock-in models are described in Tables S3-S7, which are organized by RASopathy.

In this Review, we discuss the pathogenesis of the RASopathy-associated genetic variants (Table 1; Fig. S1) and the knowledge gained about RAS/MAPK signaling that resulted from studying RASopathies. We also describe the cell and animal models of the RASopathies (Fig. 1) and explore emerging RASopathy genes. Preclinical and clinical experiences with targeted agents as therapeutics for RASopathies are also discussed, together with the potential of therapeutics developed for RAS/MAPK-driven malignancies for the prevention or treatment of RASopathies. This article serves as an update to the excellent and comprehensive reviews on the non-NF1 RASopathies (Hernandez-Porras and Guerra, 2017; Jindal et al., 2015; Schuhmacher et al., 2017). Animal models of NF1 have recently been reviewed (Williams and Largaespada, 2020) and will thus not be discussed here.

**Table 1.**
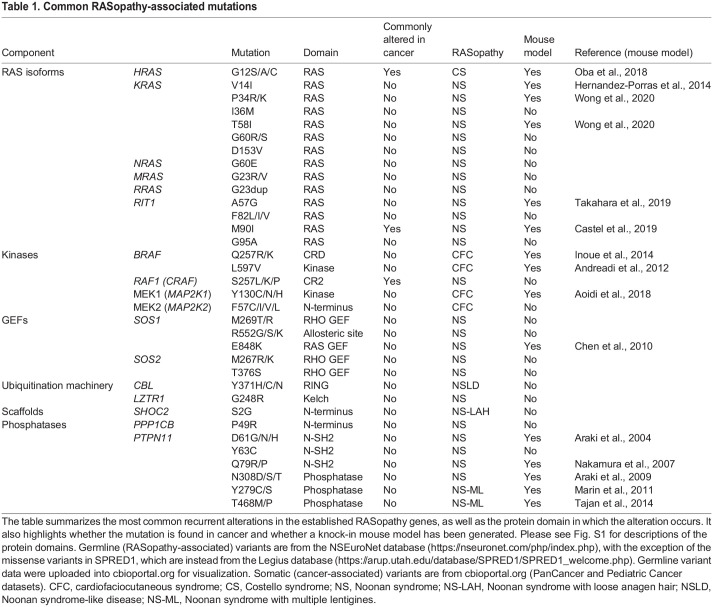
Common RASopathy-associated mutations

## NS

NS is a relatively common disorder with a prevalence of 1:1000 to 1:2500 live births (Nora et al., 1974). The most common forms of NS are inherited in an autosomal-dominant fashion; however, rare autosomal recessive forms have been described (Johnston et al., 2018; van Der Burgt and Brunner, 2000). NS is caused by germline pathogenic variants in one of several RAS/MAPK pathway genes. In particular, 50% of NS cases are caused by a gain-of-function mutation in *PTPN11*, 10% by mutation in *SOS1*, 10% by mutation in *RIT1* and 5% by mutation in *RAF1*, and an additional 5% of cases are caused by mutations in *KRAS*. The remaining 20% of cases are caused by rarer mutations in *NRAS*, *RRAS*, *LZTR1* and others (Tartaglia and Gelb, 2005).

Most patients with NS have one or more of the following clinical features: (1) facial dysmorphic features; (2) congenital heart disease; and/or (3) postnatal proportional short stature. Additional features include endocrine abnormalities, such as low body mass index, delayed puberty onset, cryptorchidism or Sertoli cell dysfunction; skeletal abnormalities or decreased bone mineral density; neurocognitive abnormalities; pulmonary lymphangiectasias; and easy bruising (Romano et al., 2010). Patients with NS are at increased risk of developing malignancies such as neuroblastoma, juvenile myelomonocytic leukemia (JMML), low-grade glioma, giant cell lesions and rhabdomyosarcoma (Romano et al., 2010). The severity of the clinical features of NS varies by genotype (Table 1). The pathogeneses of the genetic subtypes of NS are discussed below:

### PTPN11

Protein tyrosine phosphatase, non-receptor 11 (PTPN11; also known as SHP2) is a tyrosine phosphatase and molecular scaffold. Its phosphatase activity is required for full activation of the RAS/MAPK pathway. Active PTPN11 dephosphorylates many substrates (Hill et al., 2019). The cancer- and NS-associated mutations in *PTPN11* occur at the interface between the N-terminal SH2 domain at residues D62, Y63 or Q79, and in the PTP domain at residue N308, and relieve auto-inhibitory intramolecular interactions (Table 1; Fig. S1). NS-associated PTPN11 mutants thus have increased phosphatase activity (Keilhack et al., 2005; Martinelli et al., 2008; Tartaglia et al., 2006).

NS-associated *PTPN11* mutations have been studied in several model systems. Transfecting mutant *PTPN11* transgenes into mammalian cells *in vitro* increased signaling through the RAS/MAPK pathway (Fragale et al., 2004; Kontaridis et al., 2006). Zebrafish embryos expressing NS-associated *ptpn11* (also known as *ptpn11a*) mutants display increased major:minor axis ratios (Fig. 1) (Jopling et al., 2007), and adult mutants have cardiac defects that can be rescued by treatment with the MEK inhibitor CI-1040 (Bonetti et al., 2014). *Drosophila* expressing NS-associated *PTPN11* mutants display ectopic wing veins (Fig. 1) (Oishi et al., 2006) and have impaired long-term memory, a phenotype that is rescued by treatment with the pre-clinical PTPN11 inhibitor NSC-87877 (Pagani et al., 2009). Moreover, induced pluripotent stem cells (iPSCs) derived from NS patients with PTPN11^N308D^ and PTPN11^D61N^ displayed impaired early neuroectodermal development due to enhanced gliogenesis and shortened neurites. This phenotype was rescued by treatment with the preclinical compound PHPS1, a cell-permeable phospho-tyrosine mimetic that inhibits PTPN11 phosphatase activity (Ju et al., 2020). Knock-in mouse models of *Ptpn11*^N308D/+^ and *Ptpn11*^D61G/+^ (Table S3) display small body size, facial dysmorphia, splenomegaly, and deficits in spatial learning and memory (Araki et al., 2009, 2004). The *Ptpn11*^D61G/+^ mice display a variety of cardiac phenotypes, with penetrance depending on the genetic background. Intriguingly, postnatal treatment of *Ptpn11*^D61G/+^ mice with the MEK inhibitor SL327 or the HMG-CoA reductase inhibitor lovastatin rescued the observed spatial learning deficits (Lee et al., 2014), whereas postnatal treatment of *Ptpn11*^D61Y/+^ mice with the tyrosine kinase inhibitor dasatinib reversed their cardiac dysfunction (Yi et al., 2016). Studies with conditional knock-in mice have elucidated the cell of origin and developmental stage responsible for NS phenotypes. For example, tissue-specific transgenic expression of *Ptpn11*^Q79R^ in neural crest-derived tissues leads to craniofacial dysmorphia, which can be prevented with prenatal treatment with the tool compound U0126, a copper-chelating MEK inhibitor (Nakamura et al., 2009). Mice engineered to express *Ptpn11*^Q79R^ in the fetal cardiomyocytes develop ventricular septal defects and heart failure in an ERK1/2 (also known as MAPK3/1)-dependent manner. Interestingly, mice expressing the same *Ptpn11*^Q79R^ transgene in postnatal cardiomyocytes have no cardiac phenotype, underscoring that both the cell type and the developmental stage of the cell are important for this phenotype (Nakamura et al., 2007). Testing of candidate MEK, PTPN11 and SOS1 inhibitors in these models of NS could inform which NS phenotypes are reversible, which genotypes are most amenable to targeted therapy and which drugs are best tolerated.

### SOS1

Son of sevenless homolog 1 (SOS1) is a member of the RAS GEF family that activates the RAS proteins by catalyzing the exchange of bound GDP for GTP. In the basal state, the RHO GEF and PH domains of SOS1 occlude the allosteric site, preventing its full activation. A further level of auto-inhibition is achieved by the N-terminal histone-like domain interacting with the linker between the PH and REM domains (Fig. S1), locking the RHO GEF/PH domains into position (Gureasko et al., 2010; Sondermann et al., 2004). NS-associated *SOS1* mutations cluster either in the region of the RHO GEF domain that interacts with the allosteric site (M269) or in the region of the allosteric site that interacts with the histone-like domain (R552; Table 1). Both relieve auto-inhibitory interactions, increasing SOS1 GEF activity on RAS and thus signaling through the MAPK pathway (Lepri et al., 2011; Nakamura et al., 2017; Roberts et al., 2007; Tartaglia et al., 2007; Zenker et al., 2007).

Mouse models of M269- or R552-altered *SOS1* have not yet been reported. However, mice expressing *Sos1*^E846K^, an activating mutation in the GEF domain, display cardiac hypertrophy and splenomegaly (Table S3). The viability of these mice depends on the genetic background. Importantly, prenatal treatment with the MEK inhibitor PD-0325901 rescued the perinatal lethality of mice homozygous for *Sos1*^E846K^ (Chen et al., 2010). Clinical whole-exome sequencing (WES) of NS patients also identified mutations in *SOS2*, which shares 65% sequence identity with *SOS1*. These SOS2 variants cluster in a region of the RHO GEF domain that interacts with the allosteric site and lead to increased signaling through the MAPK pathway in these patients (Cordeddu et al., 2015; Lissewski et al., 2021; Yamamoto et al., 2015).

### RAF1

**Fig. 2.**
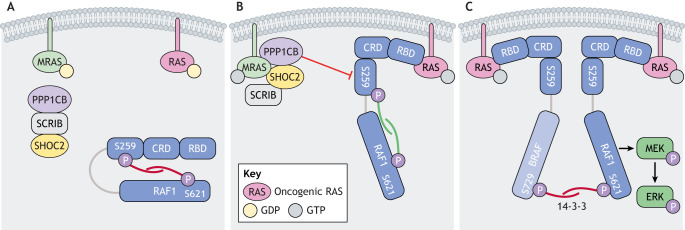
**Pathogenesis of Noonan syndrome with loose anagen hair (NS-LAH) and related disorders.** (A) In the basal state, MRAS and HRAS/KRAS/NRAS (depicted as RAS) are GDP bound, and RAF1 is held in the autoinhibited confirmation by 14-3-3 binding to phospho-serines 259 and 621. (B) The NS-LAH-associated SHOC2^S2G^ causes aberrant myristylation and membrane localization of SHOC2. Activation of MRAS, which can occur via guanine nucleotide exchange factor activity or via mutation, myristylation of SHOC2, or mutation of the PPP1CB phosphatase, facilitates the formation of a complex between MRAS-GTP and PPP1CB, SHOC2 and SCRIB. RAF1 membrane localization via binding to RAS-GTP puts RAF1 in proximity to the MRAS/SHOC2/PPP1CB complex, which allows PPP1CB to dephosphorylate S259 on RAF1. (C) In the active state, S259-dephosphorylated RAF1 is now able to homo- or heterodimerize with other RAF proteins, activating its kinase activity and triggering the signaling through MEK to ERK. 14-3-3 binding to S621 stabilizes the dimerization.

RAF1 (also known as CRAF) is a member of the RAF family of serine/threonine kinases and a RAS effector. In the basal state, the N-terminal autoinhibitory region of RAF1, composed of the RAS-binding and cysteine-rich domains (Fig. S1), occludes the kinase domain (Fig. 2). This autoinhibitory conformation is stabilized by the binding of a 14-3-3 dimer, an interaction mediated by phosphorylation of RAF1 at S259. Activated RAS recruits RAF to the plasma membrane, disrupts the auto-inhibitory binding between the N-terminal regulatory domain and C-terminal kinase domain, and induces RAF dimerization, all of which activates its kinase activity (Terrell and Morrison, 2019). Active RAF phosphorylates and activates MEK1 (also known as MAP2K1) and MEK2 (also known as MAP2K2), which in turn phosphorylate and activate ERK1 and ERK2. Both cancer- and NS-associated RAF1 mutations cluster around S259 (Table 1). These variants (e.g. S257L) are constitutively dephosphorylated at the inhibitory S259 phospho-site, leading to constitutive activation (Molzan et al., 2010; Pandit et al., 2007; Razzaque et al., 2007).

The biological effects of NS-associated *RAF1* mutations have been studied in several models. Cardiomyocytes derived from *RAF1*^S257L^ iPSCs (iPSC-CMs) display increased surface area and myofibrillar disarray. Importantly, treatment of these myocytes with the MEK inhibitors PD98059 or trametinib resolved the myofibrillar disarray but did not reduce the cell surface area (Jaffre et al., 2019), while treatment with U0126 reduced both the cell surface area and the myofibrillar disarray. Mouse models of S257-altered RAF1 have not yet been reported; however, a knock-in *Raf1*^L613V^ mouse model displays a variety of NS-like phenotypes, including cardiac hypertrophy and an indolent myeloproliferative neoplasm (Table S3). Importantly, treatment with the MEK inhibitor PD-0325901 (mirdametinib) at weaning reversed the observed cardiac defects in this model (Holter et al., 2019).

### RAS superfamily

HRAS, KRAS and NRAS are the ‘founding’ members of a larger superfamily of at least 35 related human proteins, which also includes MRAS, RRAS and RIT1 (Colicelli, 2004). RAS proteins function as binary, guanine nucleotide-regulated, on-off switches. In normal quiescent cells, RAS is predominantly cytosolic, GDP bound and inactive. Growth factors activate RAS GEFs to promote nucleotide exchange and form active RAS-GTP at the plasma membrane. Membrane localization is required for the activation of RAS proteins. This localization is mediated by a combination of C-terminal farnesylation, palmitoylation and electrostatic interactions (Cox et al., 2015). Once in the active, GTP-bound conformation, membrane-bound RAS can bind to a variety of effectors, including the RAF and PI3 kinases, to transmit downstream signals (Pantsar, 2020).

The RAS proteins were first identified in transforming retroviruses and are mutated in 30% of human cancers. In sporadic cancer, RAS proteins are typically mutated at one of three main hotspots, G12, G13 or Q61. These mutations render RAS variably GAP insensitive, thus persistently GTP bound and active (Cox and Der, 2010). By contrast, RAS mutation at A146 increases the fraction of GTP-bound activated RAS by enabling it to spontaneously exchange GDP for GTP in the absence of a RAS GEF (Janakiraman et al., 2010).

### KRAS

The RAS protein that is most commonly altered in NS is KRAS. NS-associated *KRAS* mutations are not found at the oncogenic hotspots; rather, they commonly occur at V14, P34 or T58I (Table 1; Fig. S1). In addition to these single-nucleotide variants, copy-number gain of the *KRAS* locus is also associated with NS-like phenotypes (Gilbert-Dussardier et al., 2016). Like the cancer-associated mutations, NS-associated mutations result in accumulation of GTP-bound KRAS through enhanced nucleotide exchange or impaired GTP hydrolysis, albeit to a lesser extent than the cancer-associated variants (Gremer et al., 2011; Schubbert et al., 2007).

Several mouse models of *KRAS*-associated NS have been developed (Table S3). *Kras*^V14I^ knock-in mice display a variety of NS-like phenotypes, including cardiac hypertrophy and an indolent myeloproliferative neoplasm, with the phenotypes altered by the genetic background of the model. Importantly, prenatal treatment of *Kras*^V14I^ mice with the MEK inhibitor PD-0325901 (mirdametinib) prevented perinatal lethality and cardiac defects, but postnatal treatment did not reverse these phenotypes (Hernandez-Porras et al., 2014, 2015, 2016), indicating that modulating the MAPK pathway at the right developmental time is essential. *Kras*^P34R^ and *Kras*^T58I^ mouse models of NS were recently described. Interestingly, the *Kras*^P34R^ mice have perinatal lethality due to a lung sacculation defect, while the *Kras*^T58I^ mice display cardiac hypertrophy (Wong et al., 2020). Treatment studies in these mouse models have not yet been reported.

### NRAS

Because KRAS alterations are found in NS, and HRAS alterations are typically found in CS, as discussed below, investigators performed targeted sequencing for the third ‘classical’ RAS isoform, NRAS, in patients with clinically diagnosed NS without previously known mutations. Two recurrent *NRAS* alterations were identified, T50I and G60E (Table 1; Fig. S1). Both are expected to affect the ability of NRAS to interact with effectors, GEFs or GAPs. Although *NRAS*^G60E^ is a rare somatic variant in JMML, in large part, the NS-associated *NRAS* variants are different from those identified in NRAS-altered malignancies (Denayer et al., 2012).

NS-associated *NRAS* mutants have been studied *in vitro* and *in vivo*. Overexpression studies in mammalian cells showed that each of these alterations increase signaling through the MAPK pathway. Biochemical studies showed that NRAS^G60E^ is insensitive to GAP-stimulated hydrolysis, and molecular dynamics simulations suggest that NRAS^T50I^ occupies a more signaling-competent orientation without affecting intrinsic or GAP-stimulated GTP hydrolysis (Cirstea et al., 2010). NRAS^I24N^, which was identified in a Dutch NS cohort, also increased MAPK signaling when overexpressed in mammalian cells. Importantly, the authors of this study also showed that *nras*^I24N^ and *nras*^Q60E^, but not *nras*^T50I^, cause the early developmental defect of an oval-shaped yolk sac when introduced as transgenes into zebrafish embryos, and that this could be prevented by MEK inhibition (Runtuwene et al., 2011). No mouse models of *NRAS*-mutated NS have been developed to date.

### RRAS

Protein interaction network analyses suggested that alterations in RAS-related protein (RRAS), a small GTPase that shares 48% amino acid sequence identity to HRAS, might be associated with the RASopathies. Targeted sequencing in a panel of NS patients without previously known mutations identified two *RRAS* mutations, V55M and G39dup (Flex et al., 2014). Somatic mutations in *RRAS* were also identified in the circulating leukocytes of JMML patients, including *RRAS*^G39dup^ and *RRAS*^Q87L^ (Flex et al., 2014), a mutation analogous to Q61L in the classical RAS isoforms. These mutations are all expected to affect the guanine-binding ability of RRAS.

*In vitro* biochemical studies showed that both RRAS^V55M^ and RRAS^G39dup^ displayed increased GEF-stimulated nucleotide exchange compared to the wild-type protein, and both RRAS^Q87L^ and RRAS^G39dup^ displayed decreased intrinsic and GAP-stimulated GTP hydrolysis (Flex et al., 2014; Suzuki et al., 1997). When overexpressed in mammalian cells, *RRAS*^V55M^ and *RRAS*^G39dup^ increased RAS/MAPK signaling, and transgenic *Caenorhabditis elegans* embryos expressing *ras-1*^G27dup^, the ortholog of *RRAS*^G39dup^ (Table S2), displayed abnormal vulval morphogenesis (Flex et al., 2014). Recurrent variants in the closely related *RRAS2* (also known as *TC21*) were recently identified in patients with NS. One of these, RRAS2^A70T^, displays increased GEF-catalyzed guanine nucleotide exchange and impaired intrinsic and GAP-stimulated GTP hydrolysis *in vitro*, and stimulates the RAS/MAPK pathway in mammalian cells (Capri et al., 2019). NS-associated *RRAS* and *RRAS2* variants have yet to be modeled in mice.

### RIT1

In NS patients who did not carry known NS-associated gene mutations, WES revealed recurrent mutations in the small GTPase Ras-like without CAAX 1 (RIT1; Table 1) (Aoki et al., 2013; Bertola et al., 2014; Chen et al., 2014). RIT1 is ubiquitously expressed and plays a role in neuronal survival, proliferation and differentiation. The NS-associated mutations in RIT1 are clustered in the switch II region (Fig. S1). Their expression in *Drosophila* or zebrafish models results in ectopic wing veins and major:minor axis dysmorphia, respectively (Aoki et al., 2013). Knock-in mouse models of *Rit1*^M90I^ and *Rit1*^A57G^ display facial dysmorphia, cardiac hypertrophy and splenomegaly (Castel et al., 2019; Takahara et al., 2019) (Table S3). Importantly, two infants with *RIT1*-associated NS and heart failure were treated with the MEK inhibitor trametinib and showed clinically significant improvement (Andelfinger et al., 2019), suggesting that drugs targeting the RAS/MAPK pathway might be meaningful treatment options for these patients.

### ERK2

ERK2 and its homolog ERK1 are phosphorylated downstream of MEK1/2 in the RAS/MAPK pathway. Recently, *ERK2* variants have been identified in patients with clinical features of NS (Motta et al., 2020a). Expression of these variants in mammalian cell cultures increased ERK2 translocation to the nucleus and increased the phosphorylation of ERK targets in a growth factor-dependent manner, whereas their expression in *C. elegans* caused the multi-vulva phenotype consistent with aberrant RAS/MAPK pathway activity (Motta et al., 2020a). Interestingly, the aberrant MAPK signaling observed in *ERK2* variant-expressing mammalian cells was inhibited by the MEK inhibitor trametinib (Motta et al., 2020a), indicating that MEK inhibition may be a therapeutic option for patients with *ERK2*-associated NS, despite ERK2 being downstream of MEK in the RAS/MAPK pathway.

### LZTR1

**Fig. 3.**
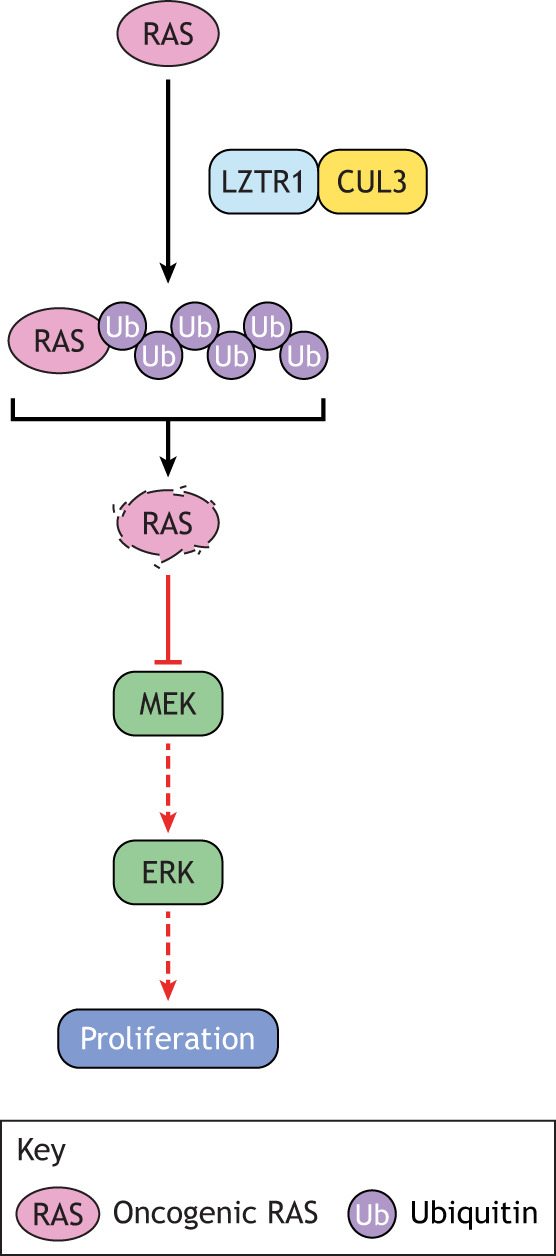
**Pathogenesis of LZTR1-associated Noonan syndrome (NS).** LZTR1 facilitates the CUL3-mediated polyubiquitination of several RAS isoforms, which leads to their degradation. Loss-of-function alterations in LZTR1, then, increase RAS stability and signaling through the RAS/MAPK pathway, driving NS phenotype.

Several WES studies identified likely pathogenic variants in leucine zipper-like transcriptional regulator 1 (*LZTR1*) in NS patients without mutations in known NS-associated genes (Johnston et al., 2018; Yamamoto et al., 2015). LZTR1, which is an adaptor for the E3 ubiquitin ligase CUL3, is recurrently altered in schwannomatosis (Piotrowski et al., 2014) and was identified as a tumor suppressor in glioblastoma multiforme (Frattini et al., 2013), and to associate with both autosomal-dominant and autosomal-recessive forms of NS. The mechanism by which LZTR1 affects the RAS/MAPK pathway was not immediately clear; however, overexpression of LZTR1 mutants such as G248R or genetic depletion of endogenous *LZTR1* in mammalian cells activated MAPK (Bigenzahn et al., 2018; Motta et al., 2019). Several groups independently discovered that LZTR1 binds to RAS family GTPases, including HRAS, KRAS, NRAS, MRAS and RIT1, facilitating their ubiquitination by CUL3 and subsequent proteasomal degradation (Fig. 3). Decreased *LZTR1* expression increases RAS stability and, thus, persistent MAPK signaling (Bigenzahn et al., 2018; Castel et al., 2019; Steklov et al., 2018). The NS-associated mutants in the Kelch domains of LZTR1 disrupt the interaction with RAS, preventing ubiquitination and degradation (Steklov et al., 2018). Deletion of *lztr1* in zebrafish causes cardiac hypertrophy (Nakagama et al., 2020), while deletion of *Lztr1* in *Drosophila* causes ectopic wing veins (Bigenzahn et al., 2018), consistent with increased MAPK signaling. In mouse models, homozygous knockout of *Lztr1* is embryonic lethal, and *Lztr1*^+/−^ mice display craniofacial, pulmonary and cardiac abnormalities (Steklov et al., 2018) (Table S3). Prenatal treatment with the MEK inhibitor pimasertib only partially reversed the embryonic lethality of *Lztr1*^−/−^ and had no effect on the pulmonary phenotype (Sewduth et al., 2020). Additionally, iPSC-CMs from NS patients with *LZTR1* alterations are larger than iPSC-CMs from unaffected individuals, suggesting that the cardiac hypertrophy observed in *LZTR1*-associated NS is linked to increased cardiomyocyte size. Treatment of *LZTR1*-mutant iPSC-CMs with the MEK inhibitor U0126 only slightly decreased their size (Hanses et al., 2020). Therefore, MEK inhibition might not be effective as a monotherapy in patients with *LZTR1*-associated NS.

### SHOC2

Alterations in the molecular scaffold SHOC2 are associated with development of the NS spectrum disorder, Noonan syndrome with loose anagen hair [NS-LAH; Online Mendelian Inheritance in Man (OMIM) 607721]. NS-LAH is most commonly caused by the S2G germline pathogenic mutation in *SHOC2* (Fig. S1). Patients with NS-LAH have easily pluckable hair, as well as NS-like facial features, growth failure, mild cognitive impairment, and cardiac defects including mitral valve and septal defects. SHOC2 forms a ternary complex with MRAS and the PPP1CB phosphatase that dephosphorylates the RAF kinases to facilitate full activation of the RAS/MAPK pathway (Young et al., 2018) (Fig. 2). The NS-LAH-associated S2G mutation causes SHOC2 myristylation and its mislocalization to the plasma membrane (Cordeddu et al., 2009). Additionally, activating mutations in MRAS (T68I, G23R/V), which augment its binding to SHOC2 and PPP1CB, have also been identified in patients with NS and hypertrophic cardiomyopathy (Higgins et al., 2017; Motta et al., 2020b), indicating a likely genotype–phenotype continuum between NS and NS-LAH.

### CBL

Alterations in the E3 ubiquitin ligase CBL are associated with development of a NS spectrum disorder (NS-like disorder with or without JMML; OMIM 613563). CBL mutations most commonly occur at Y371H. Patients with pathogenic germline *CBL* variants have subtle clinical features, like craniofacial abnormalities, café-au-lait macules, cryptorchidism, and growth and developmental delay, but a striking predisposition for JMML. The loss of the wild-type *CBL* allele via uniparental disomy is commonly observed in the JMML clone. *CBL* mutations associated with this NS-like disorder impair its ubiquitin ligase function and consequently impair the ability of CBL to induce the degradation of cell surface receptors upon ligand stimulation, ultimately resulting in prolonged activity of RAS/MAPK signaling (Martinelli et al., 2010; Niemeyer et al., 2010; Perez et al., 2010).

In summary, NS is a disorder caused by alterations in one of an expanding list of RAS/MAPK pathway genes. The severity of symptoms and the potential treatment options vary by genotype, suggesting that genetic testing is informative for prognosis and for personalized treatment strategies for NS spectrum disorder patients.

## NS-ML

NS-ML (formerly LEOPARD syndrome; OMIM 151100) is a rare autosomal-dominant syndrome with a poorly characterized prevalence (Sarkozy et al., 2008). Patients have many of the same clinical characteristics as NS patients, with the addition of sensorineural hearing loss, multiple pigmented skin lesions (lentigines) and ocular hypertelorism. Whereas pulmonary valve stenosis is the most common congenital heart defect in NS, hypertrophic cardiomyopathy (HCM) and electrocardiogram abnormalities are more common in NS-ML. In addition, short stature is less common in NS-ML than in NS. Patients with NS-ML are predisposed to neuroblastoma and acute myelogenous leukemia (Gelb and Tartaglia, 1993). NS-ML is caused by germline pathogenic variants in PTPN11, most commonly Y279C, T468M and Q506P (Table 1). The NS-ML PTPN11 variants are therefore different from those that cause NS. NS-ML PTPN11 variants are confined to the protein phosphatase domain and decrease its activity. Importantly, nonsense and frameshift alterations in *PTPN11* are associated with metachondromatosis (OMIM 156250), an autosomal-dominant syndrome in which affected patients manifest multiple cartilage tumors (Bowen et al., 2011).

*Ptpn11* knockout in the murine germline is embryonic lethal (Yang et al., 2006) (Table S1); however, knocking out *Ptpn11* in mesenchymal progenitors results in a metachondromatosis-like phenotype rather than an NS-ML one (Yang et al., 2013), which suggests that some residual activity of PTPN11 is responsible for the NS-ML phenotype. Several other *PTPN11*-based model systems recapitulate the clinical phenotypes of NS-ML. Indeed, NS-ML-associated *PTPN11* variants activate multiple signaling pathways, including RAS/MAPK, PI3K/AKT, FAK and MEK5/ERK5 (Kontaridis et al., 2006; Yu et al., 2014), when overexpressed in mammalian cells, through mechanisms that are both dependent and independent of residual phosphatase activity. Introduction of NS-ML-associated *ptpn11* variants in zebrafish increases the major:minor axis ratio and cardiac edema in embryos (Jopling et al., 2007), and causes craniofacial dysmorphia, increased melanophore formation (Bonetti et al., 2014) and cardiac asymmetry defects (Stewart et al., 2010) in adult fish. In addition, expression of NS-ML-associated *PTPN11* variants in *Drosophila* results in ectopic wing vein and rough eye phenotypes (Oishi et al., 2009). *PTPN11*^T468M^ iPSC-CMs are larger than wild-type ones and have increased MEK phosphorylation, correlating with a potential hypertrophic state (Carvajal-Vergara et al., 2010). Knock-in mouse models of *Ptpn11*^T468M^ and *Ptpn11*^Y279C^ have also been developed (Table S4) and display small size, cardiomegaly and facial dysmorphia. The *Ptpn11*^T468M^ mice have splenomegaly, and the *Ptpn11*^Y279C^ mice have pectus abnormalities (Marin et al., 2011; Tajan et al., 2014). Postnatal treatment of *Ptpn11*^Y279C^ mice with the mTOR inhibitor rapamycin, the AKT inhibitor ARQ 092 or the tyrosine kinase inhibitor dasatinib reverses the hypertrophic cardiomyopathy, suggesting that these agents might represent therapeutic options for NS-ML patients (Marin et al., 2011; Wang et al., 2017; Yi et al., 2016). Consistent with this idea, a patient with NS-ML-associated malignant HCM caused by *PTPN11*^Q510E^ was treated with the mTOR inhibitor everolimus as a bridge to cardiac transplant and experienced improvement in heart failure severity (Hahn et al., 2015). Some patients with NS-ML may therefore require different therapies than those used to treat patients with classical NS.

## CS

Unlike NS, CS is quite rare, with prevalence estimates ranging from 1:380,000 in the UK (Giannoulatou et al., 2013) to 1:1,290,000 in Japan (Abe et al., 2012). CS is an autosomal-dominant disorder caused exclusively by pathogenic germline variants in *HRAS* (Rauen, 2007). Somatic alterations in *HRAS* have been identified in transitional cell carcinoma of the bladder, thyroid cancers, head and neck squamous cell carcinoma (Moore et al., 2020), and pediatric cancers such as embryonal rhabdomyosarcoma (Vaseva and Yohe, 2020). Unlike the NS-associated variants in genes encoding various RAS proteins, the CS-associated *HRAS* mutations overlap with cancer-associated ones (Fig. S1). In most cases of CS (80%), the causative *HRAS* variant is G12S (Table 1) (Gripp et al., 2006). Several rare G12 variants, including G12V, G12D, G12C and G12E, are associated with the development of a more-severe form of CS that is lethal in infancy (Weaver et al., 2014). In addition to single-nucleotide variants, alterations that affect the splicing of *HRAS* (Guil et al., 2003) have been reported in patients with CS (Pantaleoni et al., 2017). Almost all reported cases of CS are *de novo* (Rauen, 2007).

Infants with CS frequently have polyhydramnios *in utero* and are born prematurely but large for their gestational age. Children with CS have coarser facial features than those with NS, as well as macrocephaly, prominent foreheads, epicanthal folds, short noses and low-set, posteriorly rotated ears (Goodwin et al., 2014). Patients fail to thrive in the perinatal period due to swallowing difficulties and increased energy expenditure, and the gastrointestinal involvement progresses to include gastrointestinal reflux, oral aversion and constipation (Leoni et al., 2016). CS patients have unique dermatological abnormalities including cutaneous papillomas (Siegel et al., 2012), as well as a variety of musculoskeletal abnormalities, such as hypotonia, elbow contractures and laxity of the small joints (Leoni et al., 2021). Like NS, CS also causes congenital heart defects, including HCM, septal defects and arrhythmias (Lin et al., 2011). Patients with CS have profound cognitive deficits (Axelrad et al., 2011), as well as anatomic abnormalities of the central nervous system (Gripp et al., 2010). Importantly, they have the highest risk of developing a malignancy of any of the RASopathies patients, particularly rhabdomyosarcoma, neuroblastoma and transitional cell carcinoma of the bladder (Rauen, 2007). Although the average age at diagnosis of this bladder carcinoma in the general population is 73 years, it has been diagnosed in individuals with CS who are 20 years old or younger (Gripp, 2005). CS is therefore a classical cancer predisposition syndrome.

Several models of CS have been developed. Neuronal progenitors from *HRAS*^G12S^ iPSCs display an extended progenitor phase, increased soma size and decreased neurite outgrowth, which may contribute to the neurodevelopmental disorders associated with CS (Rooney et al., 2016). Transgenic zebrafish expressing *hrasb*^G12V^ in the germline display phenotypes consistent with CS: shortened body length, craniofacial dysmorphia, precocious ossification, myocardial hypertrophy, increased incidence of cancer, and oncogene-induced senescence in the brain and heart (Santoriello et al., 2009). The initial CS mouse model (Table S5), *Hras*^G12V−IRES-β-geo^, has a phenotype of hypertension and myocardial and kidney fibrosis, but does not develop tumors (Schuhmacher et al., 2008). A subsequent model, *CC-FR-Hras*^G12V^, has a high perinatal mortality rate and CS-like craniofacial defects, and develops benign skin papillomas and malignant angiosarcoma (Chen et al., 2009). Importantly, these mice also have a skeletal muscle myopathy, which can be reversed by postnatal treatment with the MEK inhibitor PD-0325901 (Tidyman et al., 2021). A *Hras*^G12S^ mouse model has also been established. These mice have craniofacial abnormalities, cardiomegaly, poor weight gain and impaired metabolism, consistent with CS patients (Oba et al., 2018). These mice did not develop papillomas or angiosarcomas.

In summary, the available mouse models of CS demonstrate some, but not all, of the clinical features of CS. Treatment studies in CS models suggest that patients with CS may benefit from RAS/MAPK-specific therapies, as discussed in more detail below.

## CFC

Like CS, CFC is a rare RASopathy. Its worldwide prevalence is not known, but is estimated at 1:810,000 in Japan (Abe et al., 2012). CFC is inherited in an autosomal-dominant manner and is caused by germline pathogenic variants in *BRAF* (75%), *MAP2K1* (which encodes MEK1), *MAP2K2* (which encodes MEK2) or, rarely, *KRAS* (Niihori et al., 2006; Rodriguez-Viciana et al., 2006). CFC patients have craniofacial features reminiscent of NS, congenital heart disease, thin, curly and friable hair with sparse eyebrows and eyelashes, hyperkeratotic skin and frequent hemangiomas. These patients also have short stature and fail to thrive, most often due to gastrointestinal reflux and oral aversion. Neurological involvement is common in CFC and manifests as cognitive impairment, learning disabilities, hypotonia, motor and speech delay, and epilepsy. Patients with CFC may also have an increased risk of malignancy (Pierpont et al., 2014; Rauen, 2016).

### BRAF

In cancers such as melanoma, *BRAF* is typically mutated at the V600 codon (Fig. S1), allowing for the BRAF kinase activation in the absence of both dimerization and an active RAS (Freeman et al., 2013). By contrast, the most common CFC-associated mutations in *BRAF* occur at Q257 (Table 1). This residue is predicted to be a part of both the 14-3-3 interface (Park et al., 2019) and the plasma membrane interface (Travers et al., 2018), but the precise mechanism by which Q257 mutations activate BRAF are currently unknown. BRAF^Q257R^ has increased *in vitro* kinase activity compared to wild type. Similarly, transfection of *BRAF*^Q257R^ into mammalian cells yields greater increases in MEK and ERK phosphorylation than transfection of wild-type *BRAF* (Rodriguez-Viciana et al., 2006).

The biological consequences of CFC-associated *BRAF* mutations have been studied in several models. *BRAF*^T599R^ and *BRAF*^Q257R^ iPSC-CMs are hypertrophic and have altered calcium homeostasis. Treatment with U0126 reversed the hypertrophic phenotype (Josowitz et al., 2016). Expression of *BRAF*^Q257R^ in zebrafish embryos increased the major:minor axis ratio, a phenotype that was prevented by treatment with the MEK inhibitor CI-1040 (Anastasaki et al., 2009). The first murine model of *BRAF*-associated CFC (Table S6) was based on a knock-in of the oncogenic *Braf*^V600E^ mutation. Although this variant is not associated with CFC, the model has been used to study how Braf activation leads to the CFC phenotype in mice. In particular, the *Braf* ^+/LSLV600E^ mice have decreased viability, splenomegaly, facial dysmorphia, small size, hyperactivity and epilepsy (Urosevic et al., 2011). Importantly, these mice also develop spontaneous lung adenomas and melanoma (Urosevic et al., 2011). The second model also involves an alteration in the kinase domain, LSL-*Braf* ^L597V^ and recapitulates the phenotype of *Braf*^+/LSLV600E^ animals; however, instead of epilepsy, the mice can have spontaneous cerebrovascular accidents. They also develop skin papillomas and intestinal polyps, but no malignant tumors. These mice have a skeletal myopathy, which is due to failed myoblast differentiation (Andreadi et al., 2012). Treating myoblasts derived from the LSL-*Braf* ^L597V^ mice with the MEK inhibitor PD-0325901 rescued myogenic differentiation (Maeda et al., 2021). A *Braf* ^Q241R/+^ mouse model has also been developed, which corresponds to *BRAF* ^Q257R^ in CFC patients. The phenotypes of these mice vary across genetic backgrounds, but in general present as decreased viability, small size, cardiomegaly, lymphangiectasia, impairments in contextual fear conditioning and decreased esophageal motility. Prenatal exposure of *Braf* ^Q241R/+^ mice to PD-0325901 prevented perinatal lethality, and prenatal exposure to the MEK inhibitor MEK162 prevented the esophageal dysmotility (Inoue et al., 2014; Moriya et al., 2015).

### MEK1/2

MEK1 and MEK2, the products of *MAP2K1* and *MAP2K2*, respectively, are dual-specificity kinases that phosphorylate ERK1 and ERK2 in the RAS/MAPK signaling pathway. MEK1/2 are the only phosphorylation targets of active RAF kinases (Roskoski, 2012). The majority of the cancer- and CFC-associated variants in MEK1/2 occur at the interface of the auto-inhibitory region, e.g. MEK2^K57N^, and the kinase domain, e.g. MEK1^Y130C^ and MEK2^E203K^ (Table 1; Fig. S1). These mutations increase the *in vitro* kinase activity (Ordan et al., 2018), and their expression in mammalian cells increases signaling through the RAS/MAPK pathway, which can be blocked by U0126 (Rodriguez-Viciana et al., 2006; Senawong et al., 2008). However, some of the CFC-associated MEK mutations confer resistance to the MEK inhibitor trametinib (Emery et al., 2017), indicating that MEK inhibitors may not always be appropriate therapeutics for patients with MEK-associated CFC, despite the preclinical finding that elongated zebrafish embryos, a phenotype caused by expression of *map2k1*^Y130C^, can be reversed by the MEK inhibitors CI-1040 and PD-0325901 (Anastasaki et al., 2009). Expression of *Dsor1*^Y130C^, the *MAP2K1* ortholog in *Drosophila* (Table S2), leads to larval cuticle deficits and ectopic wing veins (Jindal et al., 2017). In addition, a knock-in mouse model of *Map2k1*^Y130C^ has been developed (Table S6). The *Map2k1*^Y130C^ mice have cardiac and craniofacial abnormalities, increased activated astrocytes in the sensory cortex and hippocampus, as well as increased oligodendrocyte precursors in the sensory cortex (Aoidi et al., 2018). These neuropathological changes could be important for the pathogenesis of the neurological phenotypes of CFC; however, neurobehavioral testing has not yet been performed with this mouse model. No treatment studies in these mice have been reported to date.

In summary, CFC is a rare RASopathy that is caused by alterations in one of several components of the RAS/MAPK pathway. The clinical features of CFC, as well as the associated genotypes, overlap with those of NS.

## LS

LS (OMIM 611431) is a rare, autosomal-dominant disorder caused by germline pathogenic variants in *SPRED1* (Brems et al., 2007). The prevalence of LS is currently not well characterized (Brems et al., 2012), but variants in *SPRED1* are commonly identified in patients with a clinical diagnosis of NF1 who do not have detectable alterations in *NF1* (Messiaen et al., 2009). LS patients have skin manifestations, such as café-au-lait macules similar to those of NF1 patients; however, neurofibromas, which are common in NF1, are not a feature of LS. Additionally, some LS patients have dysmorphic facial features similar to those of NS and musculoskeletal features, including pectus excavatum or carinatum and unilateral postaxial polydactyly. Neurological features are also common and include learning disabilities, attention deficit hyperactivity disorder and developmental delay. Lipomas are common and malignancies such as acute myelogenous leukemia have been reported (Brems et al., 2012).

SPRED1 is a molecular scaffold that binds to the NF1 protein and recruits it to the plasma membrane, where NF1 exerts its RAS GAP function. The LS-associated *SPRED1* variants, including nonsense, frameshift and missense mutations, prevent NF1 membrane localization (Yan et al., 2020). Expression of LS-associated *SPRED1* variants in mammalian cells increased signaling through the RAS/MAPK pathway (Brems et al., 2007).

*Spred1* knockout mice are viable but display airway hyper-responsiveness and facial dysmorphia (Denayer et al., 2008; Inoue et al., 2005). Neurobehavioral testing demonstrates that *Spred1* knockout mice have impaired spatial learning and memory (Borrie et al., 2021a), as well as altered social and communicative behavior (Borrie et al., 2021b) (Table S7). Importantly, postnatal MEK inhibition with PD-0325901 does not affect the spatial learning and memory phenotypes but does rescue the altered social behavior phenotypes (Borrie et al., 2021a,b). Knockout of the related *Spred2* results in an obsessive-compulsive behavioral phenotype (Ullrich et al., 2018), as well as impaired growth, cardiac hypertrophy, splenomegaly and craniofacial malformation (Ullrich et al., 2019). Interestingly, a recent report described a case series of patients with an autosomal-recessive NS-like phenotype resulting from loss of SPRED2 function (Motta et al., 2021), further validating *SPRED2* as a RASopathy gene.

LS, therefore, is a RASopathy with clinical features similar to NF1. The similarity between these disorders is likely to be due to the dependence of NF1 on SPRED1 for GAP activity (Stowe et al., 2012). Treatment studies in LS animal models indicate that some, but not all, of the features of LS may be ameliorated by MEK inhibition.

## CCLA

Germline alterations in several RAS/MAPK genes have been identified in patients with lymphatic anomalies. In some cases, the lymphatic anomaly is the only RASopathy-associated clinical manifestation (Sevick-Muraca and King, 2014). Recently, a recurrent alteration in *ARAF*, S214P, was identified in patients with CCLA (Li et al., 2019). This ARAF residue is analogous to S259 in RAF1, which is phosphorylated and bound by 14-3-3 in the auto-inhibited state (Fig. 2). Somatic ARAF alterations at S214 are also found in lung adenocarcinoma (Imielinski et al., 2014). Expressing ARAF^S214P^ in mammalian cells decreases the association with 14-3-3 and increases ERK phosphorylation (Li et al., 2019). Additionally, human primary dermal lymphatic endothelial cells expressing ARAF^S214P^ have increased lymphangiogenic capacity compared to wild type, a phenotype that is reversed by treatment with the MEK inhibitor trametinib (Li et al., 2019). Expression of *araf*^S214P^ in zebrafish leads to dilated lymphatic vessels, which can be reversed by treatment with the MEK inhibitor cobimetinib (Li et al., 2019). Moreover, a patient with CCLA associated with *ARAF*^S214P^ was treated with trametinib on a compassionate-use basis, which improved pulmonary function and decreased oxygen requirement, indicating meaningful clinical improvement (Li et al., 2019).

## CM-AVM

CM-AVM is an autosomal-dominant disorder affecting 1:100,000 people of northern European origin (Revencu et al., 2008). Approximately 50% of CM-AVM cases are caused by pathogenic germline variants in *RASA1*, which encodes the p120-RAS GAP (Eerola et al., 2003; Revencu et al., 2008), with the remaining caused by other RAS/MAPK pathway genes such as *EPHB4* (Amyere et al., 2017). The defining characteristic of CM-AVM syndrome is the presence of multifocal cutaneous capillary malformations, with or without concurrent arteriovenous malformations in the skin, muscle, bone or internal organs, although some patients also have cardiac defects and may also have an increased risk of cancer (Boon et al., 2005). Morpholinos targeting both zebrafish *RASA1* alleles, *rasa1* (also known as *rasa1a*) and *rasa2*, cause an embryonic vascular defect marked by an increase in mTOR signaling, which was rescued by treatment with the PI3K/mTOR inhibitor NVP-BEZ235 (Kawasaki et al., 2014). In mice, homozygous knockout of the RAS GAP domain exons of *Rasa1* either in the germline or exclusively in the blood vessel endothelial compartment is lethal at mid-gestation due to the resulting vascular defects (Lapinski et al., 2007, 2012; Lubeck et al., 2014). Treatment studies have not yet been reported in these mouse models.

## SYNGAP1 syndrome

SYNGAP1 syndrome is an autosomal-dominant disorder caused by germline loss-of-function alterations in *SYNGAP1*, which encodes a RAS GAP that is part of a subfamily that also includes DAB2IP, RASAL2 and RASAL3. The exact prevalence of the disorder is unknown, but germline *SYNGAP1* alterations are found in 0.5-1% of children with neurodevelopmental disorders. The clinical features of SYNGAP1 syndrome include global developmental delay, hypotonia, seizures, skeletal abnormalities, strabismus, constipation, failure to thrive and autism spectrum disorders (Parker et al., 2015). Homozygous knockout of *Syngap1* in mice is embryonic lethal; heterozygous mice display profound neurobehavioral changes (Nakajima et al., 2019). Both increased RAS/MAPK signaling and long-term potentiation impairment were observed in the hippocampal slices from heterozygous mice, but treatment with the MEK inhibitor PD-0325901 did not improve the long-term potentiation impairment (Kopanitsa et al., 2018).

## Emerging RASopathy genes

Next-generation sequencing has identified several genes that were altered in patients with RASopathies; however, the functional validation of these as RASopathy-associated genes is not yet complete. These include *RASA2*, *SPRY1*, and *MAP3K8* (Chen et al., 2014). Here, we discuss RASopathy genes that have been identified or validated since this topic was originally defined (Tidyman and Rauen, 2016a,b).

KAT6B (also known as MYST4) is a histone H3 lysine 23 (H3K23ac) acetyltransferase. The epigenetic implications of H3K23 acetylation are not well studied. A balanced translocation predicted to cause loss of KAT6B function was identified in a patient with NS. In addition to decreased H3K23 acetylation, cells with decreased expression of *KAT6B* show increased signaling through the RAS/MAPK pathway. Mice that are homozygous for a hypomorphic mutation in *Kat6b* show features reminiscent of NS: failure to thrive, craniofacial dysmorphia and brain abnormalities (Kraft et al., 2011). However, alterations in *KAT6B* are also observed in several other developmental disorders with features similar to those of the RASopathies, including Say-Barber-Biesecker-Young-Simpson syndrome (OMIM 603736) (Campeau et al., 2012), so further validation is needed.

Individuals with 6p-interstitial deletion syndrome (OMIM 612582) have NS-like clinical features, such as short stature, intellectual disability, craniofacial dysmorphia and cardiac abnormalities. The RAS-responsive element-binding protein 1 (*RREB1*) gene is deleted in individuals with this syndrome. RREB1 is a transcription factor that binds to RAS-responsive elements in the promoters of genes encoding components of the MAPK pathway, such as *HRAS*, *MAP2K2* and *FGFR4*. Promoter-bound RREB1 represses the expression of these MAPK pathway genes in part through its recruitment of the CtBP1 transcriptional co-repressor complex. *Rreb*^+/−^ mice are viable and display short stature, craniofacial dysmorphia and cardiac hypertrophy. Increased *Hras* expression and increased ERK phosphorylation are observed in cardiac tissue from these mice. These results suggest that *RREB1*, like *KAT6B*, may function as a RASopathy gene through a transcriptional regulation mechanism (Kent et al., 2020).

Recurrent alterations in the RHO family GTPase CDC42 have recently been identified in patients with clinical features of NS. These alterations cause vulvar phenotypes consistent with aberrant RAS/MAPK activity when expressed in *C. elegans* (Fig. 1), although their direct impact on ERK phosphorylation has not yet been assessed (Martinelli et al., 2018). This observation opens up the possibility of RASopathy-causing variants in other subfamilies of GTPases. Importantly, variants in another RHO family GTPase, RAC1, are associated with developmental delay (Reijnders et al., 2017).

The 14-3-3 family of proteins play a role both in RAF auto-inhibition maintenance and in their dimerization (Fig. 2). Recently, a germline variant in 14-3-3zeta (*YWHAZ*) was identified in a patient with a clinical diagnosis of CFC. Expression of this variant in *Xenopus tropicalis* increased BRAF and RAF1 binding, ERK phosphorylation and decreased body length, consistent with *YWHAZ* functioning as a RASopathy gene (Popov et al., 2019).

The genes discussed here have been identified as altered in small subsets of patients. In addition to these, several genes encoding components of the RAS/MAPK pathway also merit further study, even though they have not yet been identified as RASopathy genes. As new candidate RASopathy genes continue to emerge, existing RASopathy clinical sequencing panels may need to be expanded.

## Targeting aberrant RAS signaling in the RASopathies

Recently, the U.S. Food and Drug Administration (FDA) approved the MEK inhibitor selumetinib for the treatment of some patients with NF1, but no treatments are currently available for patients with non-NF1 RASopathies. In several of the mouse or other model system studies described above, a MEK inhibitor was used as a prenatal preventative therapy or postnatal treatment, raising the possibility that MEK inhibitors may be effective in the treatment of non-NF1 RASopathies. For example, in the *Sos1*^E846K^ and *Kras*^V14I^ mouse models of NS (Chen et al., 2010; Hernandez-Porras et al., 2014) and the *Braf*^Q241R^ mouse model of CFC (Inoue et al., 2014), MEK inhibitors given prenatally effectively prevented the manifestations of the modeled RASopathy. MEK inhibition also reversed RASopathy phenotypes in *PTPN11*- and *NRAS*-associated zebrafish models of NS (Bonetti et al., 2014; Runtuwene et al., 2011), *BRAF*- and *MAP2K1*-associated zebrafish models of CFC (Anastasaki et al., 2009), and *ARAF*-associated zebrafish models of CCLA (Li et al., 2019). Excitingly, MEK inhibitors were effective in treating some, if not all, of the other RASopathy manifestations, in the *Ptpn11*^D61G^ and *Raf1*^L613V^ knock-in mouse models of NS (Lee et al., 2014; Wu et al., 2011).

However, there are some murine models in which MEK inhibition had no effect on the RASopathy phenotype. MEK inhibitors were not effective when given prenatally to the *Lztr1* knockout model of NS (Sewduth et al., 2020) and the *Syngap1* heterozygous knockout mouse model of SYNGAP1 syndrome (Kopanitsa et al., 2018). In addition, despite the fact that prenatal administration prevented RASopathy phenotypes, postnatal administration did not ameliorate the phenotypes of *Kras*^V14I^ knock-in NS mice (Hernandez-Porras et al., 2014), indicating how important the timing of the intervention may be. Responses to MEK inhibition in *Spred1* knock-out mouse models of LS underline the importance of careful preclinical studies, because although the social behavior phenotypes of these mice were affected by MEK inhibition, the spatial learning and memory phenotypes were not (Borrie et al., 2021a,b). Most excitingly, however, patients with *RIT1*-associated NS and HCM had clinical improvement in their heart failure symptoms when treated with trametinib (Andelfinger et al., 2019), providing the strongest evidence to date that MEK inhibition may be an effective treatment strategy for patients with RASopathies.

Other drug classes are also effective in models of RASopathies, particularly NS-ML. As discussed above, postnatal treatment of NS-ML mouse models with inhibitors of the PI3K/mTOR/AKT pathway or with the tyrosine kinase inhibitor dasatinib reversed cardiomyopathy phenotypes (Marin et al., 2011; Wang et al., 2017; Yi et al., 2016), and treatment of a NS-ML patient with the mTOR inhibitor everolimus provided clinical benefit (Hahn et al., 2015). Treatment of a *PTPN11*-associated NS mouse model with dasatinib also reversed cardiomyopathy (Yi et al., 2016). PI3K/mTOR inhibitors are also effective in a *RASA1*-associated CM-AVM zebrafish model (Emery et al., 2017). Inhibition of other pathways, then, may be more beneficial than inhibition of the RAS/MAPK pathway in some RASopathies, although this hypothesis has yet to be formally tested.

**Fig. 4.**
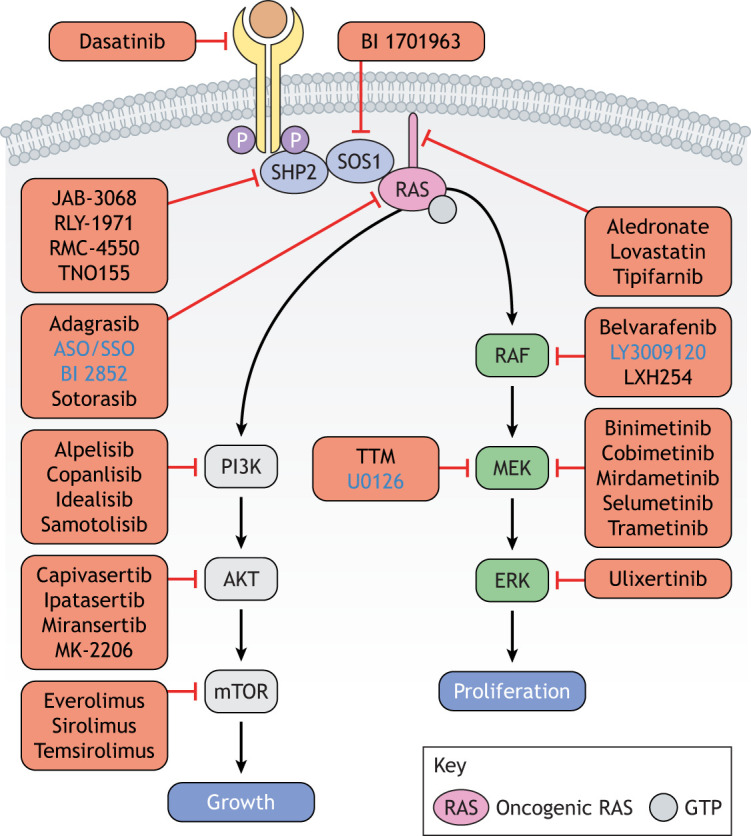
**Treating RASopathies.** Researchers have developed several classes of drugs that target RAS activation and components of the MAPK or the PI3K/AKT pathway. Drugs that inhibit RAS activation include those that target RAS directly (e.g. sotorasib), inhibit RAS membrane localization (e.g. tipifarnib), prevent the activity of SHP2 (e.g. RMC-4550), inhibit the interaction of RAS with its exchange factor SOS1 (e.g. BI 1701963), or block the kinase activity of receptor tyrosine kinases (e.g. dasatinib). Please see the text for more details. Blue font indicates preclinical tool compounds; black font indicates a drug in clinical development. Several have been FDA approved for the treatment of cancer, including sotorasib, trametinib and copanlisib, among others, and could potentially be used for the treatment of RASopathies. TTM, tetrathiomolybate.

Recent work by the National Cancer Institute's RAS initiative, industry and academic laboratories has yielded a large number of novel agents for the treatment of cancers driven by RAS/MAPK pathway alterations (Moore et al., 2020). These agents could represent treatment options for patients with RASopathies and fall into three classes: inhibitors of RAS activation, direct RAS inhibitors and inhibitors of the MAPK pathway (Fig. 4).

### Inhibitors of RAS activation

Inhibitors of RAS activation include compounds that inhibit RAS prenylation or its interactions with proteins necessary for its activation, such as SHP2 and SOS1. As discussed above, C-terminal prenylation is required for RAS membrane localization and, thus, activation. Both the statins, e.g. atorvastatin and lovastatin, and the N-bisphosphonates, e.g. alendronate and pamidronate, are metabolic inhibitors that prevent the synthesis of prenylation substrates. Either statins or N-bisphosphonates, then, might prevent RAS prenylation and, thus, its activation in patients with RASopathies (Baranyi et al., 2020). These drugs are well studied and relatively well tolerated for the treatment of chronic conditions such as hypercholesterolemia and osteoporosis, respectively, and, thus, represent potential long-term treatment options for adult and pediatric patients with RASopathies. Importantly, lovastatin reverses learning defects in the *Ptpn11*^D61G/+^ mouse model of NS (Lee et al., 2014), but it is not clear if this response is associated with a decrease in RAS activation. However, based on these results, a phase 3 clinical trial of a statin for treatment of growth and bone abnormalities in children with NS is currently underway (NCT02713945). The statins have not been studied in other RASopathy models. By contrast, the farnesyl transferase inhibitors (FTIs), such as tipifarnib, directly inhibit the enzyme responsible for farnesylation, a type of prenylation. NRAS and KRAS bypass the requirement for farnesylation by undergoing geranyl geranylation, an alternative post-translational modification that also allows for membrane localization. However, HRAS, the RAS isoform affected in CS, cannot be alternatively modified, and, thus, its membrane localization and cellular function are suppressed by FTIs (Cox et al., 2015). FTIs may therefore represent a potential therapeutic approach for CS patients specifically.

Additional targeted agents that prevent RAS activation have recently been identified. BI 1701963, which inhibits the interaction between RAS and SOS1 to inhibit RAS activation, is in clinical development for solid tumors harboring a KRAS mutation, but it also represents a potential RASopathy therapeutic (Hillig et al., 2019), although it has not yet been tested in RASopathy models. Allosteric inhibitors of wild-type PTPN11 that hold the phosphatase in the auto-inhibited confirmation and therefore limit signaling through the RAS/MAPK pathway are currently in clinical trials for cancer (Yuan et al., 2020). These drugs, which include RMC-4630, have not yet been tested in RASopathy models, but are promising potential therapeutics for all RASopathies with aberrant MAPK activity except for *PTPN11*-associated NS, because these inhibitors have decreased binding affinity for variant *PTPN11*.

### Direct RAS inhibitors

Direct inhibition of RAS proteins was considered impossible until the development of several direct covalent inhibitors of KRAS^G12C^, including sotorasib (AMG 510) (Hong et al., 2020), which has recently been FDA approved for non-small cell lung cancer harboring KRAS^G12C^. These drugs have not yet been tested in children and are likely to have limited utility in individuals with RASopathies, mainly because KRAS^G12C^ is uncommon in the RASopathies (Sebastian et al., 2021). Conversely, BI 2825, a preclinical tool compound that inhibits KRAS regardless of a particular mutation, represents an exciting potential therapeutic for RASopathy patients (Kessler et al., 2019). Agents that modify RAS expression have also been developed. These include cell-permeable antisense oligonucleotides that decrease expression of *KRAS* (Ross et al., 2017) and splice-switching oligonucleotides that induce the skipping of exon 2 in the maturation of *HRAS* mRNA, yielding an N-terminally truncated protein unable to bind guanine nucleotides (Hartung et al., 2016). These oligonucleotides have not yet been tested clinically or in RASopathy models.

### RAS/MAPK pathway inhibitors

The MEK inhibitor selumetinib is the first FDA-approved treatment for patients with a RASopathy, in particular, pediatric patients with NF1 who have symptomatic, inoperable plexiform neurofibromas (Gross et al., 2020b). The clinical benefit of selumetinib in NF1 has inspired the preclinical testing of MEK inhibitors in non-NF1 RASopathies, as discussed above. However, in addition to MEK inhibitors, other agents inhibit signaling through the RAS/MAPK pathway. For example, the MEK1/2 kinases require copper as a cofactor for their kinase activity. Copper chelation with ammonium tetrathiomolybdate, which is used clinically for individuals with Wilson disease, might represent a novel method by which to decrease MAPK signaling in individuals with RASopathies (Brady et al., 2017). Indeed, the tool compound U0126, which inhibits MEK in part due to its ability to chelate copper, is effective in reversing RASopathy phenotypes in iPSCs derived from patients with RAF1-associated NS and in BRAF- and MEK-associated CFC, as discussed above.

Several pan-RAF inhibitors are in clinical development, including LXH254 (Monaco et al., 2021) and belvarafenib (Yen et al., 2021). These drugs inhibit MAPK signaling in the absence of BRAF^V600E^, while the BRAF^V600E^-specific inhibitors, such as vemurafenib, paradoxically activate the MAPK pathway if administered in the absence of this mutation (Peng et al., 2015). ERK inhibitors, such as ulixertinib, are also in clinical development in pediatrics and may benefit individuals with RASopathies (Sullivan et al., 2018). The pan-RAF and ERK inhibitors also merit testing in RASopathy models.

Taken together, the preclinical evaluation of targeted agents specific for the RAS/MAPK pathway in models of RASopathies has revealed that MEK inhibitors show therapeutic promise. The key remaining challenges to be investigated in RASopathy model systems include careful characterization of which particular features of which RASopathies might be reversed with MEK inhibition. Thorough preclinical testing of novel RAS/MAPK targeted agents could also yield treatment options for patients.

## Conclusions

In the past several years, next-generation sequencing has enabled the identification of several new RASopathy genes, limiting the number of RASopathy cases without a genetic diagnosis. However, the expansion of patient genotyping has introduced some complexity into the discussion of what defines a RASopathy. For example, mosaic alterations affecting the RAS/MAPK pathway have been identified in congenital melanocytic, keratinocytic epidermal and sebacceous nevi. This discovery led to these birthmarks being termed ‘cutaneous mosaic RASopathies’ (Hafner and Groesser, 2013). Although these disorders are not established RASopathies like those discussed above, patients with cutaneous mosaic RASopathies are at risk of developing RAS-associated malignancies, and, furthermore, established RASopathies may occur in mosaicism. Patients with cutaneous mosaic RASopathies may therefore benefit from being included in RASopathy treatment protocols.

Defining RASopathy genes is similarly challenging. The generally accepted definition of a RASopathy gene is a gene involved in the RAS/MAPK pathway that is altered in the germline of a patient diagnosed with a RASopathy. Typically, in model systems, the expression of the RASopathy-associated variant leads to aberrant RAS/MAPK signaling (Fig. 1). However, some recently identified RASopathy genes have challenged this definition. For example, *A2ML1*, a gene altered in a patient with a clinical diagnosis of NS (Chen et al., 2014), encodes a protease inhibitor and component of the complement cascade of the immune system. This protein has no known link to the RAS/MAPK pathway and functional validation of the variants have yielded conflicting results, such that its role as a RASopathy gene has been questioned (Brinkmann et al., 2021). In addition, WES in a family affected by hereditary pancreatic cancer revealed a nonsense mutation in the gene encoding the small GTPase Rab-like protein 3 (RABL3), resulting in a truncated protein. Pancreatic cancer is not a RASopathy-associated malignancy; however, zebrafish models of hereditary pancreatic cancer with homozygous truncating mutations in *rabl3* displayed stunted growth, craniofacial dysmorphia, abnormal swimming behavior and decreased bone mineralization consistent with a RASopathy-like phenotype (Nissim et al., 2019), raising the possibility that *RABL3* is a RASopathy gene. Rigorous and systematic functional validation of candidate RASopathy genes is therefore essential for the development of accurate RASopathy diagnostic sequencing panels.

Currently, many patients receive a RASopathy diagnosis from prenatal genetic testing. In the majority of cases, the identification of a germline pathogenic variant in a RASopathy gene reveals not only the patient's genotype, but also the clinical diagnosis. For *PTPN11*, the specific variant can distinguish between a NS and a NS-ML diagnosis. However, both NS and CFC are associated with the same *KRAS* variants. The clinical diagnosis, then, depends upon the patient's clinical course. Future work will be needed to identify cooperating genetic variants or environmental conditions to ensure the accuracy of prenatal genetic diagnosis.

Recently, there has been a dramatic increase in the number of drugs that inhibit the RAS/MAPK pathway (Fig. 4). These were originally designed for the treatment of malignancies but may provide clinical benefit for RASopathy patients. The barriers to the initiation of clinical trials for patients with RASopathies include disease rarity, inter-patient phenotypic variability, lacking knowledge of natural history without treatment, incomplete knowledge of which clinical manifestations might be treated by postnatal administration of a RAS/MAPK-targeted drug, and the need for long-term drug tolerability. The evaluation of novel therapeutics in RASopathy models is thus critical for the development of interventional clinical trials for the non-NF1 RASopathies.

The available preclinical data suggest that MEK inhibition may be an effective treatment strategy in some, but not all, RASopathies. NS-ML models are sensitive to inhibition of the PI3K/mTOR pathway (Marin et al., 2011; Wang et al., 2017), and responses to MEK inhibition were limited in some models (Borrie et al., 2021a; Hernandez-Porras et al., 2014; Kopanitsa et al., 2018; Sewduth et al., 2020). In addition, some MEK inhibitors may not be effective in MEK-associated CFC because the single-nucleotide variant associated with the RASopathy could prevent inhibitor binding (Emery et al., 2017).

There are several important questions related to the use of MEK inhibitors for patients with RASopathies that can be answered using RASopathy models, beyond predicting efficacy. For example, the various MEK inhibitors in clinical development, such as selumetinib, trametinib, binimetinib (MEK162), cobimetinib and mirdametinib (PD-0325901) (Fig. 4), have not been compared in the same model to determine which may be most effective in a particular disease context. Additionally, for postnatal dosing, a drug is usually given by gavage at weaning. An alternative, ‘MEKi-in-milk’ protocol, in which the drug is given to lactating mothers, has been employed effectively in mouse models of NF1 (Jecrois et al., 2021), and may represent a strategy to deliver MEK inhibitors at an earlier developmental stage in non-NF1 RASopathy models.

The choice of drug dose is also important. For NF1, clinical efficacy is observed at doses lower than the recommended adult cancer dose, which also do not completely abrogate ERK phosphorylation (Jousma et al., 2015). Lower doses of MEK inhibitors may therefore be effective and have greater long-term tolerability in models of non-NF1 RASopathies. The use of the novel agents discussed above (Fig. 4), which can be selected specifically for the patient's genotype, may also improve tolerability.

The ultimate goal of all of these efforts – improved genetic diagnosis, effective preclinical models and accurate drug development – is to provide meaningful therapeutic options for RASopathy patients.

## Supplementary Material

Supplementary information
