# Supplementary material for: The RASopathies: from pathogenetics to therapeutics
Source: Dis Model Mech. 2022 Feb 18;15(2):dmm049107. doi: 10.1242/dmm.049107 (PMC8862741; doi:10.1242/dmm.049107)
Supplement: Supplementary information [file dmm-15-049107-s1.pdf]

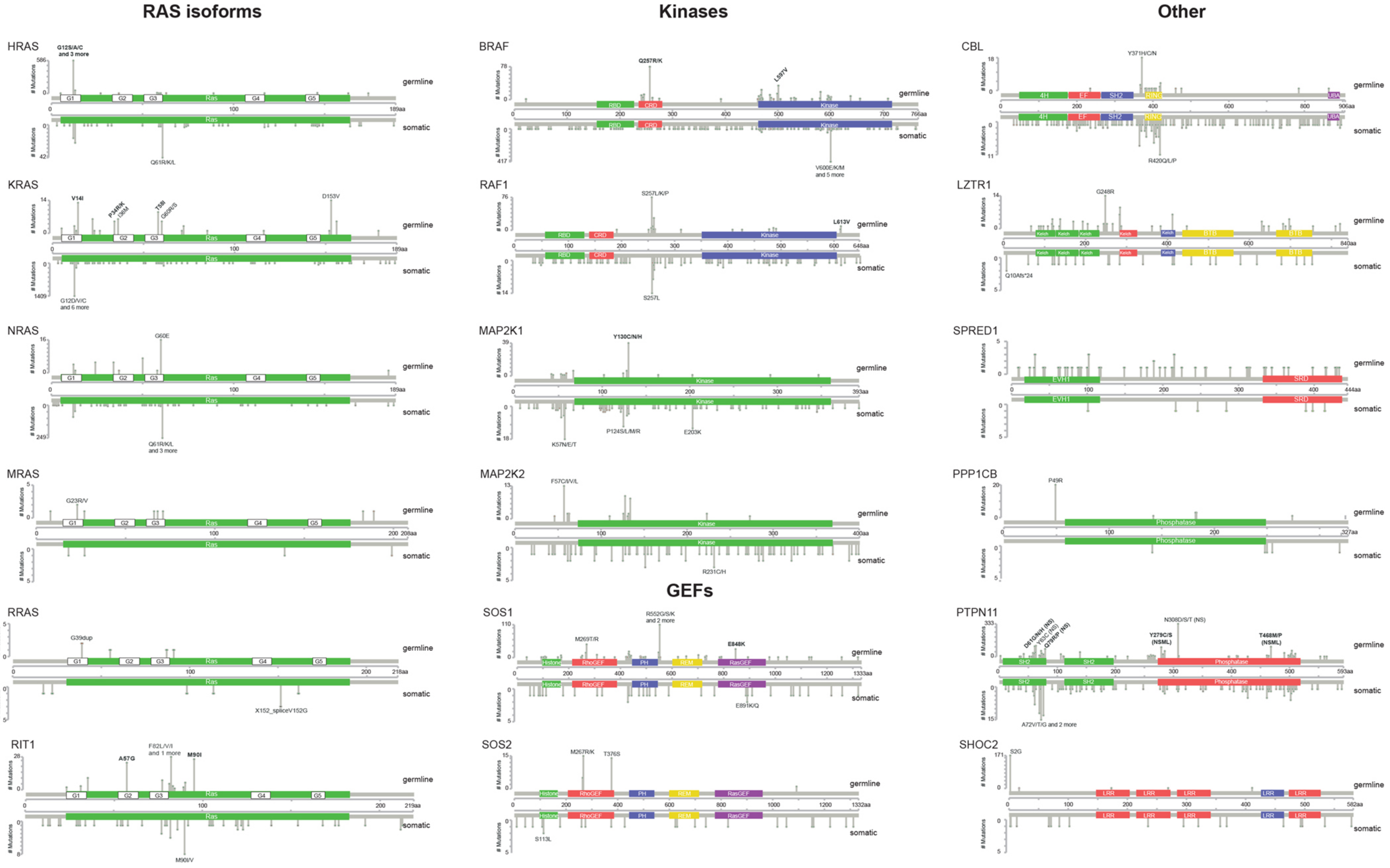

Fig. S1. Germline and somatic variants in the RAS/MAPK pathway. Germline (RASopathy-associated) variants are from the NSEuroNet database (https://nseuronet.com/php/index.php), with the exception of the missense variants in SPRED1, which are instead from the Legius database (https://arup.utah.edu/database/SPRED1/SPRED1_welcome.php). Germline variant data were uploaded into cbioportal.org for visualization. Somatic (cancer-associated) variants are from cbioportal.org (PanCancer and Pediatric Cancer datasets). In these lollipop plots, histograms of the frequency of variation at a particular amino acid are plotted on a linear representation of the domain architecture of the protein. In this figure, we are showing lollipop plots for RAS isoforms, kinases, RAS GEFs, and other components of the RAS/MAPK pathway. The RAS domain for each of the RAS isoforms is broken into 5 G-motifs, which are regions known to interact with bound guanine nucleotide. RBD, Ras binding domain; CRD, cysteine rich domain; HF, histone-like fold; PH, pleckstrin homology domain; REM, Ras exchange motif; 4H, 4 helix bundle; EF, EF hand motif; UBA, ubiquitin-associated; SH2, Src Homology 2; BTB, Broad-complex, Tram-track, Bric-a-brac; EVH1, Ena/VASP homology 1; SRD, Sprouty related domain; LRR, leucine rich region. Germline variants in bold type represent variants for which a knock-in mouse model has been created.

Table S1. Homozygous knockout mouse models of RAS/MAPK genes

Gene	Viability	Phenotype	Reference
Hras	Viable	None detected	(Esteban et al., 2001)
Kras	Embryonic lethal (E12-14)	Hematopoietic defect	(Johnson et al., 1997)
Nras	Viable	None detected	(Umanoff et al., 1995)
Hras/Nras	Viable	None detected	(Esteban et al., 2001)
Rit1	Viable	None detected	(Cai et al., 2011)
Rras	Viable	None detected	(Komatsu and Ruoslahti, 2005)
Rras2	Viable	Abnormal B and T cell survival	(Delgado et al., 2009)
Mras	Viable	None detected	(Nunez Rodriguez et al., 2006)
Araf	Perinatal lethal	Abnormal proprioception and aganglionic megacolon	(Pritchard et al., 1996)
Braf	Embryonic lethal (E10.5-E12.5)	Vascular defects	(Wojnowski et al., 1997)
Raf1 (Craf)	Embryonic lethal (E13.5)	Vascular defects	(Huser et al., 2001)
Araf/Raf1	Embryonic lethal (E10.5)	Vascular defects	(Mercer et al., 2005b)
Map2k1 (Mek1)	Embryonic lethal (E10.5)	Vascular defects	(Giroux et al., 1999)
Map2k2 (Mek2)	Viable	None detected	(Belanger et al., 2003)
Mapk3 (Erk1)	Viable	Abnormal T cell maturation	(Pages et al., 1999)
Mapk1 (Erk2)	Embryonic lethal (E11.5)	Placental defects	(Hatano et al., 2003)
Sos1	Embryonic lethal (E9 – E11)	Placental defects	(Qian et al., 2000)
Sos2	Viable	None detected	(Esteban et al., 2000)
Rasa1	Embryonic lethal (E9.25-E10.5)	Abnormal yolk sac vascularization	(Henkemeyer et al., 1995)
Syngap1	Perinatal lethal	Decreased synaptogenesis	(Kim et al., 2003)
Lztr1	Perinatal lethal	Decreased weight, facial dysmorphia, congenital heart disease	(Steklov et al., 2018)
Shoc2	Embryonic lethal	Hypoplastic endocardial cushions	(Yi et al., 2010)
Spred1	Viable	Facial dysmorphia, impaired spatial learning and memory, abnormal social behavior	(Inoue et al., 2005)
Spred2	Viable	Facial dysmorphia, splenomegaly, cardiac hypertrophy, small size	(Ullrich et al., 2019)
Spred1/Spred2	Embryonic lethal (E12.5-15.5)	Impaired lymphatic vessel development	(Taniguchi et al., 2007)
Ppp1cb	Perinatal lethal	Decreased body weight	(Meehan et al., 2017)
Ptpn11	Embryonic lethal (E3.5-E6.5)	Abnormal trophoblast proliferation	(Yang et al., 2006)

Disease Models & Mechanisms • Supplementary information

Table S2. Orthologs of RAS/MAPK genes. Orthologs obtained from https://www.flyrnai.org/cgi-bin/DRSC_orthologs.pl.

H. sapiens	*M. musculus*	*C. elegans*	*D. melanogaster*	*D. rerio*
HRAS	*Hras*	*let-60*	*Ras85D*	*hrasb*
KRAS	*Kras*	*let-60*	*Ras85D*	*kras*
NRAS	*Nras*	*let-60*	*Ras85D*	*nras*
RIT1	*Rit1*	*NA*	*Ric*	*rit1*
RRAS	*Rras*	*ras-1*	*Ras64B*	*rras*
MRAS	*Mras*	*ras-2*	*Ras64B/Ras85D*	*mras*
ARAF	*Araf*	*lin-45*	*Raf*	*araf*
BRAF	*Braf*	*lin-45*	*Raf*	*braf*
RAF1	*Raf1*	*lin-45*	*Raf*	*raf1b*
MAP2K1	*Map2k1*	*mek-2*	*Dsor1*	*map2k1*
MAP2K2	*Map2k2*	*mek-2*	*Dsor1*	*map2k2a*
SOS1	*Sos1*	*sos-1*	*Sos*	*sos1*
SOS2	*Sos2*	*sos-1*	*Sos*	*sos2*
RASA1	*Rasa1*	*gap-3*	*vap*	*rasa1a*
SYNGAP1	*Syngap1*	*gap-2*	*CG42684*	*syngap1b*
CBL	*Cbl*	*sli-1*	*Cbl*	*cbl*
LZTR1	*Lztr1*	*F53E4.1*	*Lztr1 (CG3711)*	*lztr1*
SHOC2	*Shoc2*	*soc-2*	*Sur-8*	*shoc2*
SPRED1	*Spred1*	*unc-34*	*Spred*	*spred1*
PTPN11	*Ptpn11*	*ptp-2*	*csw*	*ptpn11a*
PPP1CB	*Ppp1cb*	*gsp-1*	*flw*	*ppp1cb*

Disease Models & Mechanisms • Supplementary information

Table S3. Germline mouse models of NS. In this table and those that follow, ND denotes not determined, WNL denotes within normal limits

	**Ptpn11$^{D61G/+}$**	**Ptpn11$^{N308D/+}$**	**Sos1$^{E846K/+}$**
Strain	B6 X 129Sv	B6	B6
Viability	Decreased viability (background specific)	viable	Viable, decreased life expectancy
Cardiac	Septal defects	WNL	Aortic stenosis, cardiomegaly
Pulmonary	ND	ND	ND
Hematologic	Splenomegaly, MPN	Splenomegaly, MPN	Splenomegaly, MPN
Craniofacial	Facial dysmorphia	Facial dysmorphia	Facial dysmorphia
Neurologic	Deficits in spatial learning and memory	Deficits in spatial learning and memory	ND
Oncologic	ND	ND	ND
Dermatologic	ND	ND	ND
Musculoskeletal	ND	ND	ND
Reference	(Araki et al., 2009; Araki et al., 2004; Lee et al., 2014)	(Araki et al., 2009)	(Chen et al., 2010)

	**Raf1$^{L613V/+}$**	**Raf1$^{D486N/+}$**
Strain	B6 X 129Sv	B6 X 129Sv
Embryonic-Heterozygotes	Viable, decreased survival (B6 embryonic lethal)	Viable, decreased survival
Cardiac	Eccentric cardiac hypertrophy	Concentric cardiac hypertrophy
Pulmonary	ND	ND
Hematologic	Splenomegaly, MPN	WNL
Craniofacial	Facial dysmorphia	Facial dysmorphia
Neurologic	Astrogliosis	ND
Oncologic	ND	ND
Dermatologic	ND	ND
Musculoskeletal	ND	ND
Reference	(Holter et al., 2019; Wu et al., 2011)	(Wu et al., 2012)

Disease Models & Mechanisms • Supplementary information

	**Kras$^{V14I/+}$**	**Kras$^{P34R/+}$**	**Kras$^{T58I/+}$**
Strain	B6 X 129Sv	B6	B6 X 129Sv
Viability	Viable, fertile	Perinatal lethality	Viable
Cardiac	Cardiac hyperplasia	WNL	Cardiomegaly
Pulmonary	ND	Sacculation defect	WNL
Hematologic	Splenomegaly, MPN	ND	Splenomegaly, MPN
Craniofacial	Facial dysmorphia	Facial dysmorphia	Facial dysmorphia
Neurologic	ND	ND	ND
Oncologic	Histiocytic sarcoma, lymphoma, adenomas	Histiocytic sarcoma, lymphoma	Histiocytic sarcoma, lymphoma
Dermatologic	ND	ND	ND
Musculoskeletal	ND	ND	ND
Reference	(Hernandez-Porras et al., 2014; Hernandez-Porras et al., 2015; Hernandez-Porras et al., 2016)	(Wong et al., 2020)	(Wong et al., 2020)

	**Rit1$^{M90I/+}$**	**Rit1$^{A57G/+}$**	**Lztr1$^{+/-}$**
Strain	B6	B6	B6
Viability	Viable	Perinatal lethality (50%), decreased longevity	Decreased longevity
Cardiac	Hypertrophy	Hypertrophy	hypertrophy
Pulmonary	ND	ND	ND
Hematologic	Splenomegaly	Splenomegaly	WNL
Craniofacial	Facial dysmorphia	Facial dysmorphia	Facial dysmorphia
Neurologic	ND	ND	ND
Reference	(Castel et al., 2019)	(Takahara et al., 2019)	(Steklov et al., 2018)

Disease Models & Mechanisms • Supplementary information

Table S4. Germline knock-in mouse models of NS-ML

	**Ptpn11$^{Y279C/+}$**	**Ptpn11$^{T468M/+}$**
Strain	B6	B6 X 129Sv
Viability	Viable, decreased survival	Viable
Cardiac	Cardiomegaly	Cardiomegaly, dilated cardiomyopathy
Pulmonary	ND	ND
Hematologic	WNL	splenomegaly
Craniofacial	Facial dysmorphia	Facial dysmorphia
Neurologic	ND	ND
Oncologic	ND	None
Dermatologic	ND	ND
Musculoskeletal	Pectus carinatum and excavatum	ND
Reference	(Marin et al., 2011)	(Tajan et al., 2014)

Table S5. Germline knock-in mouse models of CS

	**CC-FR-Hras^{G12V}**	**Hras^{G12V}-IRES-β-geo**	**Hras^{G12S}**
Strain	B6	B6	B6
Viability	Perinatal lethality (80%)	Viable	Viable
Cardiac	WNL	Hypertrophy, enlarged aortic valves	cardiomegaly
Pulmonary	ND	ND	ND
Hematologic	ND	ND	WNL
Craniofacial	Facial dysmorphia, tooth enamel defect	Facial dysmorphia	Facial dysmorphia
Neurologic	ND	Learning deficits, hyperemotivity	ND
Oncologic	Papilloma, angiosarcoma	None	none
Dermatologic	ND	ND	Atopic dermatitis
Musculoskeletal	ND	ND	ND
Reference	(Chen et al., 2009; Goodwin et al., 2014)	(Schreiber et al., 2017; Schuhmacher et al., 2008; Viosca et al., 2009)	(Katata et al., 2020; Oba et al., 2018)

Disease Models & Mechanisms • Supplementary information

Table S6. Germline knock-in mouse models of CFC

	**Braf$^{+/LSLV600E}$**	**LSL-Braf^{L597V}**	**Mek1$^{Y130C/+}$**
Strain	B6 X 129Sv	B6	129Sv
Viability	Decreased	70% survive to adulthood	Viable
Cardiac	Cardiomegaly	Cardiomegaly	WNL
Pulmonary	ND	WNL	ND
Hematologic	splenomegaly	splenomegaly	WNL
Craniofacial	Facial dysmorphia	Facial dysmorphia	Facial dysmorphia
Neurologic	Hyperactivity, seizures	ND	astrogliosis
Oncologic	Lung adenomas, melanomas	Skin papillomas, intestinal polyps	none
Musculoskeletal	ND	Skeletal myopathy	ND
Reference	(Mercer et al., 2005a; Urosevic et al., 2011)	(Andreadi et al., 2012; Maeda et al., 2021)	(Aoidi et al., 2018)

	**Braf$^{Q241R/+}$**	**Braf$^{Q241R/+}$**	**Braf$^{Q241R/+}$**
Strain	B6	BALB/c	ICR
Viability	Embryonic lethal E14.5	Perinatal lethality (90% by P28)	Perinatal lethality (50% by P28)
Cardiac	Cardiomegaly	Cardiomegaly, ASD	Cardiomegaly
Pulmonary	ND	ND	ND
Hematologic	Lymphangiectasia	ND	Lymphangiectasia
Craniofacial	ND	ND	ND
Neurologic	ND	ND	Learning deficit
Oncologic	ND	ND	ND
Musculoskeletal	ND	ND	polydactyly
Dermatologic	ND	ND	Long dystrophic nails
Reference	(Inoue et al., 2014)	(Moriya et al., 2015)	(Moriya et al., 2015)

Table S7. Germline knockout mouse model of LS

	**Spred1$^{-/-}$**
Strain	B6
Viability	Viable
Cardiac	WNL
Pulmonary	Increased airway hyperresponsiveness
Hematologic	WNL
Craniofacial	Facial dysmorphia
Neurologic	Impaired spatial learning and memory
Oncologic	ND
Dermatologic	ND
Musculoskeletal	ND
Reference	(Borrie et al., 2021a; Borrie et al., 2021b; Denayer et al., 2008; Inoue et al., 2005)

Disease Models & Mechanisms • Supplementary information
